# Professional and academic profile of the Brazilian research ethics committees

**DOI:** 10.1186/s12910-022-00847-z

**Published:** 2022-11-11

**Authors:** Eugênio Pacelli de Veras Santos, Iara Coelho Zito Guerriero

**Affiliations:** 1 FMABC, Santo André, SP Brazil; 2 Integration Faculty of Sertão, Serra Talhada, PE Brazil

**Keywords:** Research ethics committee, Research ethics board, Institutional review board, CONEP, Research ethics

## Abstract

**Background:**

Brazil is among the sixteen countries that conducts the most clinical trials in the world. It has a system to review research ethics with human beings made up by the National Commission on Research Ethics (CONEP) and 779 Research Ethics Committees (RECs), in 2017. The RECs are supposed to follow the same rules regarding their membership, although the RECs that review Social Science and Humanities (SSH) researches must respect Resolution 510/16. There are Brazilian RECs that review SSH and clinical trials. This study aimed to analyze the academic professional profile of the members of the CONEP and Brazilian RECs, their adequacy to the norms, and the challenges faced by the REC’s Chairs to compose their membership.

**Methods:**

All 779 Brazilian RECs’ chairs are invited to fill in a questionnaire informing academic and professional background of the RECs members, and 92 answered. However, eight were excluded for having sent an incomplete questionnaire, leaving a total of 84 participants. The variables were described by absolute and relative frequency. The Chi-square test and ANOVA was used to analyze regional differences related difficulties to compose the committee. The significance level was 95%.

**Results:**

The results showed a predominance of members from the biomedical area (57%), while 33% were members of the Social Sciences and Humanities and 5.5% were community representatives. As for the academic degree, there were (45.2%) PhD and (27.9%) masters. The divergences in relation to the guidelines result from the difficulties of having participants in some areas and the little interest in the work carried out by the committees.

**Conclusion:**

The RECs are partially adequate to the norms and their performance may be compromised by the low participation of community representatives. The organization of REC’s specifics to review biomedical research could improve the ethical review process, ensuring a membership more qualified for these protocols.

## Background

Brazil is among the sixteen countries that conducts the most clinical trials in the world [1], which highlights the importance of an adequate ethics review. Historically, the need for ethics review of research involving human beings led to the establishment of review groups independent from the research team—Research Ethics Committees (RECs). The International Guidelines for Biomedical Research Involving Human Beings (CIOMS 2016) indicate that the committees must have a composition capable of adequately evaluating research protocols in order to ensure the rights and well-being of research subjects [2].

The Nuremberg Code, the Declaration of Helsinki, the Belmont Report and the CIOMS (2016) have influenced regulatory standards on ethical issues [2–5]. However, although following a universal parameter, ethical review structures have been developed according to the specifics of each country.

In Brazil, the system of research ethics review was established by Resolution No. 196 of the National Health Council (CNS) from October 10, 1996 [6]. This resolution was derogated by Res 466/12, that reaffirms, among other aspects, the composition, structuring and performance of the RECs and CONEP, and established the CONEP linked to the National Health Council [7].

CONEP is a National advisory, normative, deliberative, educational and independent body. Its attributions include: proposal and updating of ethics guidelines and norms, final approval and monitoring of projects in special thematic areas, registration, accreditation and supervision of the RECs. It also acts as an instance of appeal for those involved in research with human beings [7].

According to CNS Res 446/11 the composition of CONEP is multidisciplinary, composed of 30 titular members. Twenty-two of them are chosen, through curriculum analysis, among the candidates nominated by the RECs and eight are nominated by CNS directly, and must be members of CNS, respecting these criteria: four community representatives, two health professionals and two nominated by federal government or by private health services [8, 9].

In 2017, the composition of CONEP presented thirteen men and fourteen women, with twenty members having a degree in Biomedical Sciences and six in Social Sciences and Humanities (SSH). One of the members had two degrees in the SSH area. Among them, twenty-one completed specialization, twenty-two master's degree and twenty-four were PhD. One of the members had two doctoral degrees. There was only one member with an engineering degree (doctorate level), who works as geneticist (Table 1).

**Table 1:** Titling of CONEP members. Source: authors

	Bachelor	Specialization	Master’s	PhD
Area	N	N	N	N
Biomedicine	20	16	12	17
SSH	6	5	10	6
Engineering	–	–	–	1
No degree	–	5	4	3
Not informed	1	1	1	1
TOTAL	27	27	27	28*

*One of the members had two PhD degrees

Brazil had 779 RECs in 2017, and the composition of the RECs is determined by the Operational Standard 001/2013 CNS (CNS OS 001/13). The minimum composition of the RECs is seven members, multidisciplinary, with 50% of the members having proven experience in research, with a balance of members regarding sex and without a marked predominance (more than 50%) of a professional category. The term of mandate and choice of members are determined by the internal regulations of each REC [10].

The creation and organization of RECs, as well as the choice of members, were left to the institutions. The Resolution No. 240 from CNS (CNS Res 240/97) informs that community representative should be nominated by forums or non-governmental organizations that represent end users of SUS [11]. In October 2020 this resolution was revoked by CNS Resolution 647 (CNS Res 647/20), which states that each REC must have at least two community representatives, and keep one community member per seven members. And the community representative was renamed as research participant representative [12].

An accredited REC is certified by CONEP to review high risk protocols involving human beings, including all clinical trials conducted in Brazil, and the standards for the accreditation process were established in the CNS Resolution No. 506 of 2016. Among the requirements to obtain the accreditation certificate, the REC must have at least one member with curriculum experience in bioethics or research ethics in its board and prove the effective and continuous participation of the community representative in the last three years [13].

In 2016, CNS Res 510/16 established guidelines for research in the social sciences and humanities. In order to review these protocols, RECs must have a composition with equal representation of members of the SSH, and the rapporteurs must be chosen from among them [14].

The objective of the study was to analyze the academic professional profile of the members of the CONEP and Brazilian RECs, their adequacy to the norms, and analyze the challenges faced by the RECs’ Chairs to compose their membership. It is fundamental to define the decisions taken by the RECs, which has important implications on the quality of the opinions, including to clinical trials’ deadlines.

## Methods

A cross-sectional study with a quantitative approach carried out with Brazilian RECs’ chairs, identified on the CONEP website, in September 2017.

### Research stages

E-mails were sent to the REC, inviting their chair to participate in the study and including: an e-mail address to access the informed consent and the questionnaire to be answered by the chairs; a standard form to be filled out with data on academic and professional background of the members. After thirty days, the same material was sent by mail to the RECs, with the possibility of answering by mail without cost, so there was the option to participate in this way. After sending e-mails and correspondence, telephone contact was made with the RECs that did not respond to reiterate the invitation. The collection period lasted from September to December 2017.

### Instruments

The data were collected using information provided by the REC and the questionnaire answered by the chair, containing ten closed-ended questions in which respondents could choose more than one answer. A database was built with the information obtained using EXCEL software in Portuguese. The data were analyzed quantitatively by descriptive statistics and presented in matrices according to analyzed items.

### Data analysis

Two databases were created in the Microsoft Excel 2010 program—one for REC information and one for the questionnaires answers. The data were analyzed, compared with the literature and legislation in force.

The variables were described by absolute and relative frequency. The ANOVA and Chi-square test and was used to analyze regional differences, time of participation in the REC and related difficulties to compose the committee. The significance level was 95%. The statistical program used was Jasp Version 12.0.

## Results

In the analyzed RECs, participation time averaged 46.09 (+ 38.72) months and there were a high number of women (85.4%). There were many members participating in the committees for a long time and another part with little REC time. Only 5.5% of members were identified as community representatives, and 48.2% had never participated in another REC. Most of the RECs in this study were from the Northeast, 36.9% (N = 31), followed by the South with 28.6% (N = 24) and the Southeast with 14.3% (N = 12). Out of the members (N = 1118), 45.2% had completed a doctorate; 27.9% had completed a master's degree and 9.8% had completed a specialty; 2.4% of the members had only high school education (Table 2).

**Table 2:** Information on the RECs. Source: Authors

		No	%
Members’ degrees	High school	27	2.4
	Bachelor	42	3.7
	Specialization	110	9.8
	Master’s	312	27.9
	Doctorate	505	45.2
	Post-doctorate	84	7.5
Sex	Male	163	14.6
	Female	955	85.4
Has been a member of another REC	YES	50	4.4
	NO	539	48.2
Community Representative	YES	61	5.5
	NO	508	45.4
	Missing Values	549	49.1
Members by Region	Center-west	107	7.9
	Northeast	463	41.5
	North	91	8.1
	Southeast	209	18.7
	South	248	22.2

**Fig. 1:**
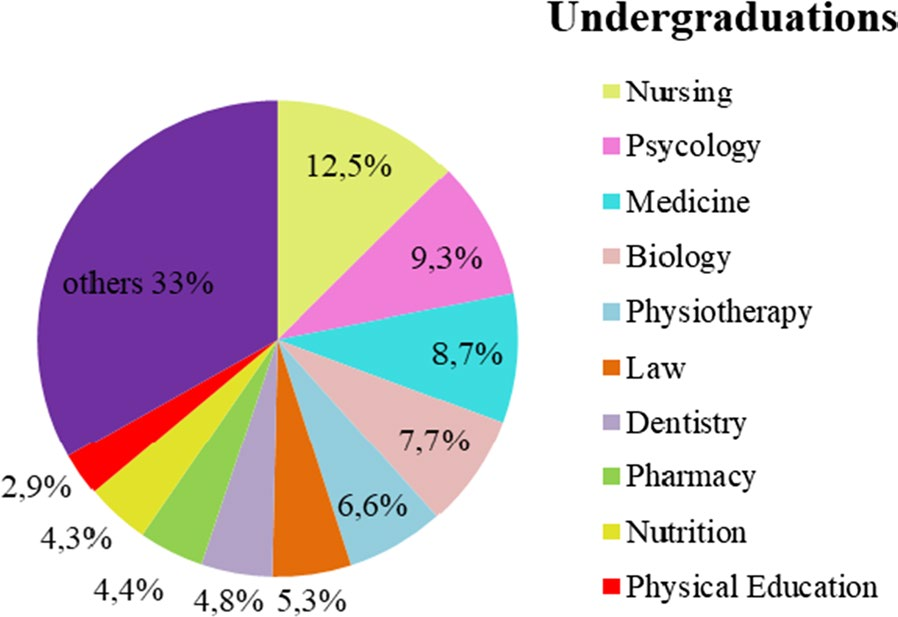
The most common degree among the RECs was a bachelor degree. Source: Authors

Of the 73 identified under graduations (degrees), 57% (N = 617) were in the biomedical area; 33% (N = 358) in the social sciences and humanities and 7% (N = 65) were distributed in the remaining areas. High school graduates represented only 3%. Nursing, psychology and medicine were the most prevalent professions—12.5, 9.3, 8.6%, respectively (Fig. 1).

The answers to the questionnaires are shown in Table 3.

**Table 3:** Questionnaire’s answers. Source: Authors

Question	Answer	N	%
Who defines the members	Institution	32	38.1
	Members	16	19
	Institution + Member + Coordinator	12	14.3
Criteria for defining the members	Time availability	16	19
	Research experience	13	15.5
	Time availability and research experience	25	29.8
	Knowledge on the REC/CONEP system	2	2.4
	Research experience and bioethics	2	2.4
Hard to get	Community representative	41	48.8
	Social Sciences and Humanities	13	15.5
	Community and Social Sciences and Humanities	12	14.3
Difficulties to participate in the REC	Lack of time	29	34.5
	Lack of time and institutional incentive	12	14.3
	Lack of time and interest	8	9.5
	No score on curriculum (associated values)	21	28.8
Adapting the REC’s composition to the standards	Easy. The rules are compatible with the institutional and professional reality of the REC	28	33.3
	Difficulty to find member for some categories	22	26.2
	Difficulty due to lack of interest or possibility to participate in the REC	15	17.9
Meeting the deadlines of CNS OS 001/13	Easy. Deadlines compatible with the REC’s composition and profile	49	58.3
	Difficulty to meet deadlines in the analysis of specific areas	14	16.7
	Difficult. The deadlines are not compatible with the number of REC members	8	9.5
Composition suitable for accreditation	Yes	34	40.5
	No	13	15.5
	The REC is not interested in the accreditation	15	17.9
	The REC is adapting itself for the accreditation process	14	16.7
Suiting the composition to CNS Res 506/16	Easy. The rules are compatible with the institutional and professional reality of the REC	28	33.3
	Difficult. The rules are not compatible with the institutional and professional reality of the REC	22	26.2
	Difficulty due to the professionals’ lack of interest or possibility of participating in the REC	13	15.5
	Operational and financial difficulty	3	3.6
Suitable for reviewing SSH research	Yes	64	76.2
	No	8	9.5
	The REC is still adapting itself to review projects in this field	7	8.3
Suiting the REC’s composition to the CNS Res 510/16	Easy. The rules are compatible with the institutional and professional reality of the members	50	59.5
	Operational difficulty. The rules are not compatible with the institutional and professional reality of the members	13	15.5
	Difficulty due to the professionals’ lack of interest or possibility of participating in the REC	11	13.1

## Discussion

The present study involved 84 RECs’ chairs. Some RECs, including those from public universities, did not participate on the grounds of data confidentiality, although information on composition is considered public. In countries such as Japan, China, Belgium and the United Kingdom, the disclosure of the names of REC’s members is usually determined [15–20]. The name of the chair and the participating REC were kept confidential and only the participants who agreed to the informed consent had access to the questionnaire.

Even though the rate response was low, the findings were not affected because the challenges for the composition of the REC are the same regardless of the location and size of the REC.

Regarding the prolonged participation in the REC of some members, there is no normative impediment in the CNS resolutions and it can be considered an advantage, since experienced members make revisions more agile. However, there is difficulty in keeping committee members for longer periods, especially the community representative, a fact observed by other works carried out in Brazil and USA [21, 22].

Even though there was a predominance of members of the biomedical area (57%), the percentage values are proportional and without hegemony of any profession, reflecting greater participation of the various categories. The higher proportion of nursing professionals differs from other studies conducted in Brazil and the number of degrees found indicates greater diversity and multidisciplinary in the RECs, a fact that raises the level of participation and understanding perspectives of the projects reviewed [21, 23]. However, this diversity may increase the difficulty to review clinical trials.

The high number of PhD (45.2%) and masters (27.9%) among REC members can be a credibility factor before the community [24], on the other hand, the hegemony of professionals with high degrees may inhibit the performance of members with less scientific knowledge, such as community representatives [22, 25].

All participating RECs had a community member, however the low participation (of members who identified themselves as) community members (5.5%) may be due to their low performance. The high number of missing values seen in Table 3 may indicate fear in disclosing that user representation is not being carried out in an ideal way and the data on participants who have only high school education (2.4%) may indicate that there are RECs in which community representation is carried out by people with higher education level.

The deliberations of a REC without the effective participation of the community member are compromised because this member represents the community's perspective on the analyzed research.

Reaching and maintaining community representatives are difficulties present in RECs and there are cases in which they are appointed only to comply with a regulation of the REC, having no distinguished action. The low participation of these members is a deficiency in the intended democratization of the committees and compromises the RECs role of social control idealized for the Brazilian system of ethical review [21, 23]. The approval of CNS Res 647/20, which states that each REC must have at least two research participant representatives [12], may result in an even more challenging situation.

The difficulty of the community representative is also observed in the Klitzman study carried out in the USA. Iijima et al. found that the external member of some RECs in Japan belonged to the institution and other committees did not even have the community member.

The disproportion with prevalence of female (85.4%-Table 2) differs from other studies [21, 23] and indicates the need for future investigations to elucidate this finding. It may represent some conjuncture beyond the issue of sex and is a condition that can influence the decisions of RECs. On the contrary, the low participation of women was observed in the ethics committees of Iran [26] and Japan [27] where there were even committees without their presence.

The number of members per region (Table 2) was influenced by the lack of information provided by the RECs. Since the Southeast of Brazil has a larger number of educational and research institutions, and a larger number of RECs, it was expected that more participants would come from this region; however, only 18.7% were from the Southeast RECs. The low participation of RECs can be seen as a lack of interest or fear in exposing the existing reality. Anyway, this behavior is strange coming from institutions that must ensure ethics in research.

The indication of members by the institution (38.1%) and by other members of the REC (19%) is the same as in India [28]. In Brazil, members are appointed by the institution that houses the REC, in other countries such as Italy [29] and France [30] the nominations of members of ethics committees occur through regional entities such as health districts.

The lack of time cited by participants as the main difficulty for professionals to participate in RECs justifies the option "availability of time" as the main criterion for selecting members (Table 3). The lack of institutional incentive is also in line with other study [23], on the other hand, it is at odds with the regulations in our country [10], which require commitment from the institution to ensure minimum operating conditions for the REC and to provide resources for continuous maintenance and investment in personnel and infrastructure.

Resistance or little interest in being a member of a REC may occur because it requires knowledge about a huge variety of research methodologies and the guidelines on research ethics. In addition, the profile of volunteer work is a cause of dissonance among researchers, but the voluntary nature of the members may influence the performance and interest in participating in the RECs. The volume and complexity of research protocols, especially clinical trials, result in an additional workload, with many hours spent by qualified professionals, which may require a full-time dedication to the committee, making it difficult to demand voluntary work [31, 32].

The origin of the RECs-CONEP System directed to biomedical sciences [33, 34] and the finding that 39.3% of RECs are located in institutions in this specific area, such as hospitals and biomedical teaching and research institutions, justifies the majority of members belonging to this segment, however 15% of the committees presented inadequate composition, with more than 80% of biomedical area members, even though their coordinators considered ease to adapt to CNS Res 510/16. This is an aspect that must be improved in Brazil, perhaps establishing specifics RECs to review only Biomedical research.

The lack of incentive from the institution, as a factor of difficulty to align the composition of the RECs to the resolutions of the CNS, manifested only by 2.4% of the coordinators (Table 2), may demonstrate that the participant did not want to expose weaknesses in the system, considering it inconvenient to disclose institutional information, despite the commitment of confidentiality present in the study.

The objective of REC accreditation is that it can perform reviews of research involving more risk, including clinical trials that will be realized inside or outside the institution, issuing the final opinion, when forwarded by CONEP through the Brazil Platform. Although 40.5% of participants (Table 2) consider that the REC in which they participate has an adequate composition for the accreditation process, it is observed that those who are in the process of adapting to apply for the accreditation certificate will face challenges to compose their boards—either due to lack of time, or lack of interest from potential candidates for membership.

The lack of interest or possibility of professionals to participate in the committee was more highlighted by the participants than operational and financial difficulties. This reality externalized by the coordinators may reflect a greater awareness of the institutions on the importance of the REC's presence or, at least, the normative need to have a well-structured ethics committee.

The increase of work influences the performance of the committees, as seen in question six, in which it was informed that the insufficient number of members made it difficult to meet deadlines. The overload of work for REC members is identified in studies by other authors [35, 36] and the lack of interest in the accreditation certificate, admitted by 17.9% of the participating chairs, may also be because this process means additional work.

Of the 64 RECs (76.2%) that the coordinators found to have adequate composition to review SSH projects, 24 did not meet the requirements of CNS Res 510/16, and the difficulty of meeting deadlines in protocol review in specific areas (16.7%) may be due to problems in obtaining members for the SSH. In addition, SSH members were the second most difficult category to obtain participants for the REC, so the opinion of 76.2% chairs (Table 2) may be due to lack of knowledge about CNS Res 510/16. The biomedical training of 54.8% of the chairs may also have influenced it, indicating that this result may be related to lack of knowledge about SSH methodologies and the methodological and ethical aspects involved in these projects.

For most of the participants (59.5%), it is easy to adapt the composition of the REC to CNS Res 510/16, but this opinion does not fit with the information contained in the undergraduate profile (Fig. 1) and the challenges cited (Table 2) to obtain representatives of the SSH.

In the present study, no significant associations were found between time of participation in the REC, regions of the country and difficulties faced. The challenges such as: how to choose members, criteria to define them, difficulties in community and SSH members, time and interest in participating, institutional incentive, among others, are not current and are present in the opinion of coordinators from all regions of the country, regardless of the time of participation in the REC. It is possible that the means used to overcome the challenges have not been efficient, and the possibility that little has been done cannot be ruled out either.

Regional particularities such as socioeconomic differences, number of educational institutions, number of committees and researchers, among others, seem not to influence the composition of the REC.

## Conclusion

The RECs-CONEP system has high education level members with many masters and doctorates. The committees’ profile is partially adequate to the interdisciplinary foreseen in the CNS resolutions. Currently RECs have a majority of members from the biomedical area and low participation from other areas. The role of the RECs may be compromised by the limited participation of community representatives.

The RECs-CONEP System is well structured, analyzes projects from all areas of knowledge and, unlike other countries that have their systems regulated by law, is standardized through resolutions enforced by National Health Council (CNS).

## Data Availability

The data-sets used and/or analyzed during the current study are available from the corresponding author on reasonable request. This is subject to participants’ expressed permission to share transcript of the information.

## Abbreviations

CNS: National Health Council
CNS Res: Resolution of the National Health Council
CNS OS: Operational standard of the National Health Council
CONEP: National Comission on Research Ethics
REC: Research ethics committee
SSH: Social science and humanities
SUS: Unified Healt System
